# SIRT5 promotes the osteo-inductive potential of BMP9 by stabilizing the HIF-1α protein in mouse embryonic fibroblasts

**DOI:** 10.1016/j.gendis.2025.101563

**Published:** 2025-02-18

**Authors:** Lu Liu, Fanglin Ye, Yue Jiang, Wenting Liu, Dongmei He, Wenge He, Xiang Gao, Hang Liu, Junyi Liao, Baicheng He, Fang He

**Affiliations:** a Department of Nephrology, The First Affiliated Hospital of Chongqing Medical University, Chongqing 400016, China; b Department of Pharmacology, School of Pharmacy, Chongqing Medical University, Chongqing 400016, China; c Key Laboratory of Biochemistry and Molecular Pharmacology of Chongqing, Chongqing Medical University, Chongqing 400016, China; d Department of Bone and Soft Tissue Oncology, Chongqing University Cancer Hospital, Chongqing 400030, China; e Department of Orthropetics, The First Affiliated Hospital of Chongqing Medical University, Chongqing 400016, China; f Department of Orthropetics, The Second Affiliated Hospital of Chongqing Medical University, Chongqing 400016, China

**Keywords:** BMP9, HIF-1α, Osteogenic differentiation, Post-translation modification, SIRT5

## Abstract

Bone morphogenetic protein 9 (BMP9) exhibits remarkable osteogenic potential. However, the intricate mechanisms driving this function of BMP9 remain elusive. This study endeavors to investigate the potential role of sirtuin 5 (SIRT5) in enhancing BMP9's osteogenic capacity and decipher the underlying molecular pathways. To achieve this aim, we employed real-time PCR, western blotting, histochemical staining, and a cranial defect repair model to assess the impact of SIRT5 on BMP9-mediated osteogenesis. We utilized real-time PCR, western blotting, immunofluorescent staining, and immunoprecipitation assay to explore the associated mechanisms. Our results revealed that SIRT5 significantly up-regulated BMP9-induced osteogenic markers, while SIRT5 knockdown reduced their expression. Concurrently, hypoxia-inducible factor 1 subunit alpha (HIF-1α) level was increased by SIRT5, but reduced by SIRT5 knockdown. Notably, HIF-1α potentiated the SIRT5's ability to strengthen BMP9's osteogenic potential, whereas HIF-1α silencing reduced this effect, which was confirmed by bone defect repair assay. The acetylation and malonylation levels of HIF-1α were reduced by SIRT5, which may enhance its stability to promote BMP9's osteogenic effect. Conversely, SIRT5 knockdown reversed these effects and promoted the degradation of HIF-1α. Collectively, our results demonstrated that the BMP9's osteogenic potential could be promoted by SIRT5, potentially through stabilizing HIF-1α by reducing its acetylation and malonylation modification. This discovery may offer a novel strategy to accelerate bone tissue engineering by enhancing osteogenic differentiation, and it also sheds light on the possible mechanisms underlying BMP9-mediated osteogenic differentiation.

## Introduction

Bone morphogenetic proteins (BMPs) encompass a diverse range of physiological functions. BMP2 and BMP7 have garnered clinical recognition for their utilization as alternatives to autografts in the treatment of bone defects and related disorders.^1^ Recently, BMP9 has been demonstrated a stronger osteogenic induction potential compared with BMP2 and BMP7,^2^ which holds promise as a growth factor for bone tissue engineering. Nevertheless, there remain certain drawbacks associated with BMP9, including the simultaneous occurrence of adipogenesis and the lengthy duration required for bone maturation.^3^ Hence, there is a need to augment the osteogenic potential of BMP9 to align with the advancements in bone tissue engineering. In addition to the BMP/Smad pathway, various other signaling pathways and factors have been discovered to be implicated in the regulation of BMP9's osteogenic potential, including the Wnt/β-catenin pathway, the retinoic acid signaling pathway, and cyclooxygenase-2 (COX-2).^4^, ^5^, ^6^ Nevertheless, the precise mechanism underlying the osteogenic potential of BMP9 remains elusive.

Hypoxia-inducible factors (HIFs) are transcription factors that respond to low oxygen conditions, which can direct cells to perceive and adapt to such environments, thereby protecting them from damage and ensuring survival.^7^^,^^8^ This family comprises several members, with hypoxia-inducible factor 1 (HIF-1) being highly conserved and expressed in oxygen-breathing organisms. HIF-1 exists as a heterodimer, comprised of two subunits, HIF-1α and HIF-1β.^9^ The two subunits share structural similarities, yet the transcriptional activity of HIF-1 primarily relies on the abundance of HIF-1α due to its short half-life, which is tightly regulated through post-transcriptional mechanisms.^10^ Previous research has revealed that BMP9 can elevate the protein levels of HIF-1α, and osteogenic differentiation induced by BMP9 can be enhanced by HIF-1α, which may be due to promoted angiogenesis or augmented glycolytic flux via up-regulation of pyruvate dehydrogenase kinase 1 (PDK1).^11^^,^^12^ However, the exact mechanisms underlying how BMP9 regulates the expression and function of HIF-1α remain elusive.

Sirtuins are NAD^+^-dependent signaling proteins that participate in diverse metabolic regulation processes, including their roles as deacetylases, desuccinylases, and demalonylases.^13^ To date, seven members of the sirtuin family have been identified (SIRT1–7). SIRT5 is predominantly located in mitochondria and possesses enzymatic activities such as desuccinylase, deacetylase, and demalonylase. Thus, the biological functions of SIRT5 are intimately related to the regulation of metabolic processes, anti-inflammatory responses, anti-cancer, and oxidative stress adaptation.^14^ In mitochondria, SIRT5 primarily regulates biological processes such as ammonia metabolism, fatty acid oxidation, glycolysis, and the tricarboxylic acid cycle. However, SIRT5 is also presented in the cytoplasm and is predominantly involved in modulating the pentose phosphate pathway and glycolytic pathways.^14^ Under hypoxic conditions, the expression level of SIRT5 in osteoblasts is reduced; overexpression of SIRT5 can effectively inhibit hypoxia-induced reactive oxygen species generation and mitochondrial depolarization, thereby mitigating osteoblast apoptosis.^15^ SIRT5 can also inhibit osteoclast formation by desuccinylating cell division cycle 42 (CDC42).^16^ Our previous study also demonstrated that SIRT5 could promote BMP9-induced osteogenic differentiation. Besides, SIRT5 could promote the proliferation and metastasis of breast cancer by increasing aerobic glycolysis, which may be mediated by up-regulating HIF-1α.^17^ However, it remains unclear whether HIF-1α is involved in the effect of SIRT5 on enhancing the osteogenic ability of BMP9.

Therefore, in this study, we aimed to investigate the potential role of SIRT5 in osteogenic differentiation induced by BMP9 in mouse embryonic fibroblasts (MEFs), and to elucidate whether HIF-1α is involved in this biological process.

## Materials and methods

### Cell culture and chemical reagents

MEFs were extracted from NIH mouse embryos as reported.^18^ HEK293, C3H10T1/2, C2C12, MC3T3-E1, and MC3T3-L1 were ordered from the American Typical Cultures Collection (Manassas, VA, USA). The medium used for cell culture contained 10% fetal bovine serum, 100 μg/mL streptomycin, and 100 U/mL penicillin. Cells were incubated at 37 °C and 5% CO_2_. Primary antibodies against RUNX family transcription factor 2 (RUNX2; sc-390351), osteopontin (OPN; sc-21742), and HIF-1α (sc-13515) were purchased from Santa Cruz Biotech (Santa Cruz, CA, USA); primary antibodies against SIRT5 (67257-1-Ig) and ubiquitin (10201-2-AP) were purchased from Proteintech (Wuhan, China); primary antibodies against acetyllysine (PTM-105RM) and malonyllysine (PTM-901) were purchased from PTM BIO (Hangzhou, China); primary antibody against β-actin (AC038) was purchased from ABclonal (Wuhan, China).

### Construction of recombinant adenovirus

Using the Ad-Easy system, recombinant adenoviruses carrying BMP9, SIRT5, and HIF-1α coding sequences were engineered and tagged with green fluorescent protein (GFP). Additionally, recombinant adenoviruses for siRNA oligonucleotides targeting HIF-1α and SIRT5 were constructed and labeled with red fluorescent protein. These constructs were designated as AdBMP9, AdSIRT5, AdHIF-1α, AdsiHIF-1α, and AdsiSIRT5, respectively. A recombinant adenovirus expressing GFP alone was used as the vector control.

### Total RNA extraction, reverse transcription reaction, and real-time quantitative PCR

Total RNA was extracted utilizing Trizol reagent (15596018, Invitrogen, Carlsbad, CA, USA). The quality and concentration of the RNA were assessed using a NanoDrop One spectrophotometer (Thermo Fisher Scientific, USA). Following the manufacturer's protocol for the reverse transcription kit (#R037A, Takara), 1 μg of total RNA was used to produce cDNA. Real-time quantitative PCR was then conducted using the SYBR Green Kit (B21202, Bimake, Shanghai, China) on a Bio-Rad CFX Connect System, and the program was as follows: pre-denaturation at 95 °C for 2 min, then denaturation at 95 °C for 20 s, annealing at 55 °C for 20 s, and extension at 72 °C for 40 cycles. The relative mRNA expression level of target genes was calculated with the 2^−ΔΔCt^ method, and the data were normalized to the expression of *β-actin*. The primers for quantitative PCR assay in this study are shown in Table 1.

**Table 1:** Primers used for reverse transcription-polymerase chain reaction.

Gene	GenBank entry	Primer	Sequence (5′→3′)
β-actin	NM_007393.5	F	CCACCATGTACCCAGGCATT
		R	CGGACTCATCGTACTCCTGC
Runx2	NM_001145920.1	F	GCCAATCCCTAAGTGTGGCT
		R	AACAGAGAGCGAGGGGGTAT
OPN	NM_001204233.1	F	TGCACCCAGATCCTATAGCC
		R	CTCCATCGTCATCATCATCG
HIF-1α	NM_010431.2	F	CTGGGACTTTCTTTTACCATGC
		R	AATGGATTCTTTGCCTCTGTGT
SIRT5	NM_178848.3	F	ACTCTTCCTGAAGCCCTTGC
		R	TTGGGGCTTGAAGGGTGTTT

(F: forward; R: reverse).

### Total protein extraction and Western blot analysis

Cells were seeded in 6-well plates. Subsequently, they were treated with AdGFP, AdBMP9, AdSIRT5, AdsiSIRT5, AdHIF-1α, and/or AdsiHIF-1α according to the experiment design. After 1, 2, 9, or 11 days, the medium was discarded, and cells were rinsed twice with cold phosphate-buffered saline (PBS, 4 °C). The plates were placed on ice, and 300 μL of radioimmunoprecipitation assay lysis buffer containing protease and phosphatase inhibitors was added to each well. Lysates were transferred to 1.5 mL EP tubes, centrifuged at 4 °C and 12,000 *g* for 10 min, and the supernatants were collected. To each tube, 75 μL of sample buffer was added, followed by boiling for 15 min to denature the proteins. The samples were then stored at −80 °C. Proteins were separated by sodium dodecyl sulfate-polyacrylamide gel electrophoresis and transferred onto polyvinylidene difluoride membranes. The membranes were treated with protein-free rapid blocking solution for 15–30 min, followed by three washes with tris buffered saline with Tween 20. The primary antibody was incubated at 4 °C overnight or at room temperature for 2 h. After three additional washes, the membranes were incubated with the corresponding horseradish peroxidase-labeled secondary antibody for 1 h. Following three washes with tris buffered saline with Tween 20, protein bands were visualized using a chemiluminescent kit (#160072, Saimik Biotech, Chongqing, China) on the BioRad ChemiDoc XRS + imaging system (Bio-Rad, Hercules, CA, USA). The data were quantified using ImageJ software.

### Alkaline phosphatase (ALP) staining assay

Cells were seeded in 24-well plates and subjected to treatment with AdGFP, AdBMP9, AdSIRT5, AdsiSIRT5, AdHIF-1α, and/or AdsiHIF-1α following the experimental design. On day 5 and day 7, the culture medium was discarded, and cells were washed three times with PBS. The ALP activity was determined using a specific assay kit (#C3206, Beyotime, Shanghai, China). Images of the plates were captured by scanning and photography using a microscope (IX53, Olympus, Japan). Quantitative analysis was carried out utilizing ImageJ software.

### Alizarin red S staining assay

Cells were seeded in 24-well culture plates and treated with AdGFP, AdBMP9, AdSIRT5, AdsiSIRT5, AdHIF-1α, and/or AdsiHIF-1α according to the experimental plan. On day 14 and day 21, the culture medium was discarded and rinsed with PBS. The cells were then fixed with 4% paraformaldehyde at room temperature for 20 min, followed by washing twice with PBS (pH 4.2). Subsequently, 0.4% alizarin red S working solution was added to each well, followed by incubation for 5 min. After incubation, the solution was discarded, and the cells were washed twice with PBS (pH 4.2). Finally, the plates were scanned, and images were captured using a microscope (IX53, Olympus, Japan). Quantification of the staining was performed using ImageJ software.

### Immunofluorescent staining assay

Cells were seeded in 35 mm confocal dishes and subjected to treatment with AdGFP, AdBMP9, AdSIRT5, AdsiSIRT5, AdHIF-1α, and/or AdsiHIF-1α following the experiment plan. Following a 24-h incubation, the culture medium was discarded, and the cells were washed with PBS and then fixed with 4% paraformaldehyde for 20 min, followed by washing twice with PBS. Subsequently, 0.5% Triton X-100 was added, and the cells were placed on ice for 20 min to permeabilize. Then, the cells were blocked with 5% bovine serum albumin at room temperature for 30 min. After the addition of the corresponding primary antibodies, the cells were incubated at 4 °C overnight and then washed with PBS. Subsequently, the cells were incubated with a Dylight649-conjugated secondary antibody (#A23610; Wuhan Abbkine Biotech, Wuhan, China) at room temperature for 1 h and then treated with an anti-fluorescent quenching agent (P0126, Beyotime, Shanghai, China). Finally, images were taken using a laser confocal scanning microscope (TCS-SP8SR, Leica, Germany).

### Cranial defect repair experiment

This experiment was approved by the Institutional Animal Care and Utilization Committee of Chongqing Medical University (IACUC-CQMU-2022-0021). Female C57BL/6J mice (aged 6–8 weeks, weighing 18–25 g) were procured from the experimental animal center of Chongqing Medical University. The mice were anesthetized using 2% sodium pentobarbital. Small holes were created on one side of the mouse skull using a 3 mm diameter ring drill. Cells were seeded in 24-well plates and treated with AdGFP, AdBMP9, AdSIRT5, AdsiSIRT5, AdHIF-1α, and/or AdsiHIF-1α in accordance with the experimental plan. After 48 h, the medium was discarded, and pre-cooled sterile PBS was added to each well to make the cells detach and form a membrane-like attachment. Then, the cell-membrane products (1 × 10^6^ cells) were collected and implanted into the bone defect sites within 4 h, and the wounds were subsequently sutured. After 4 weeks, the mice were euthanized, and the specimens were collected for micro-CT scanning (SkyScan1276, Bruker, Germany).

### Immunoprecipitation assay

Cells were plated in 100 mm culture dishes and treated with AdBMP9 in combination with AdSIRT5 or AdsiSIRT5. After 48 h, the medium was discarded, and the cells were washed twice with PBS. Dishes were placed on ice, and the cells were lysed using 1 mL lysis buffer (P0013, Beyotime, Shanghai, China) containing inhibitors of phosphatase and protease (#B14001, #B15001-A, #B15001–B, Biomark Biotech, USA). The lysates were collected and centrifuged at 4 °C and 14,000 *g* for 10 min. The supernatant was collected and divided into three new EP tubes for input, negative control (IgG), and immunoprecipitation groups. The pre-treated magnetic beads were added to the IgG group and immunoprecipitation group and incubated on a shaker at 4 °C for 1 h. The supernatant was collected and incubated with the corresponding primary antibody at 4 °C overnight with gentle shaking. Subsequently, 30 μL of pre-treated magnetic beads were added and incubated by shaking at 4 °C for 1 h. The supernatant was discarded and the beads were washed twice with lysis buffer. Finally, 25 μL loading buffer and 2 μL β-mercaptoethanol were added. The mixture was boiling for 15 min to denature protein, and the samples were subjected to regular Western blot assay.

### Statistical analysis

The experiments were conducted in triplicate independently. The data were presented as mean ± standard deviation. The statistical significance of differences between the two groups was evaluated using *t*-test, and that of differences among more than two groups was evaluated using ANOVA. Differences were defined as statistically significant when the *p* value was less than 0.05.

## Results

### Effects of BMP9 on SIRT5 expression in mesenchymal stem cells

**Figure 1:**
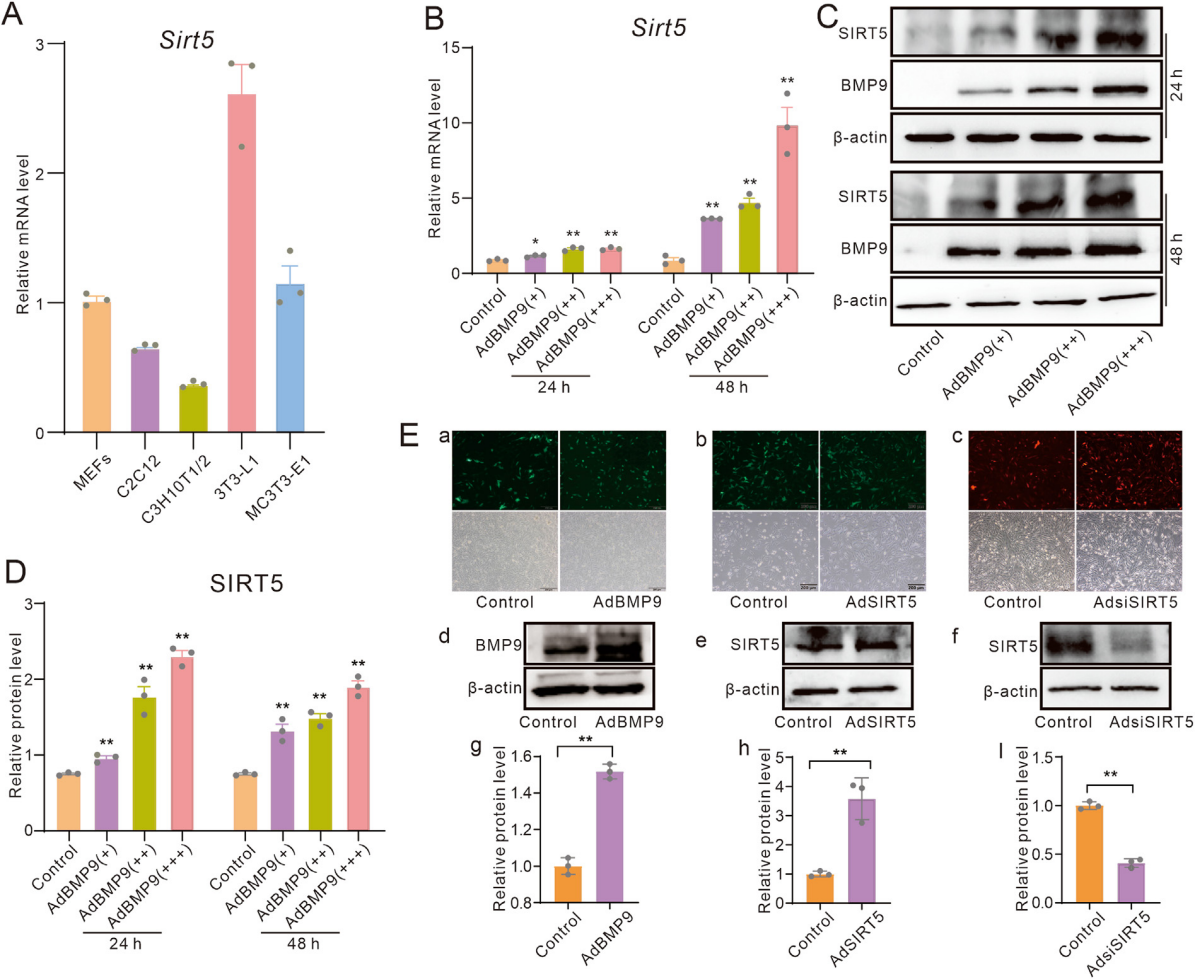
Effects of BMP9 on SIRT5 expression in mesenchymal stem cells. **(A)** Real-time PCR analysis showed the effect of BMP9 on SIRT5 mRNA expression levels in MEFs. **(B)** Western blot assays showed the influence of BMP9 on SIRT5 protein levels in MEFs. **(C)** Quantitative analysis of Western blot data highlighted the impact of BMP9 on SIRT5 protein abundance in MEFs. **(D)** Real-time PCR results showed the endogenous SIRT5 mRNA expression patterns in various progenitor cell types. **(E)** Fluorescent and phase images showed the infection results of recombinant adenoviruses (a: AdBMP9, b: AdSIRT5, c: AdsiSIRT5), Western blot results showed the effects of recombinant adenoviruses on the protein level of targets (d: AdBMP9, e: AdSIRT5, f: AdsiSIRT5), and quantitative results of Western blot assay showed the effects of recombinant adenoviruses on the protein level of targets (g: AdBMP9, h: AdSIRT5, i: AdsiSIRT5). Scale bar = 200 μm ∗*p* < 0.05 and ∗∗*p* < 0.01 versus control. BMP9, bone morphogenetic protein 9; SIRT5, sirtuin 5; MEFs, mouse embryonic fibroblasts.

While BMP9 exhibits remarkable osteogenic potential, the precise role of SIRT5 in this process remains elusive. In this study, we initially investigated the impact of BMP9 on SIRT5 expression in mesenchymal stem cells. Real-time PCR analysis showed that SIRT5 was endogenously expressed in various progenitor cell lines, with a higher level in the 3T3-L1 cell line compared with the C3H10T1/2 cell line (Fig. 1A). Given the absence of genomic modifications in MEFs, we selected it for the subsequent experiments. Real-time quantitative PCR and Western blot assay data showed that SIRT5 mRNA and protein levels could be increased by BMP9 in MEFs in a concentration-dependent manner (Fig. 1B–D). These data suggested that SIRT5 may be involved in regulating the BMP9-induced osteogenic differentiation.

### Effects of SIRT5 on the BMP9-induced osteoblastic markers in MEFs

**Figure 2:**
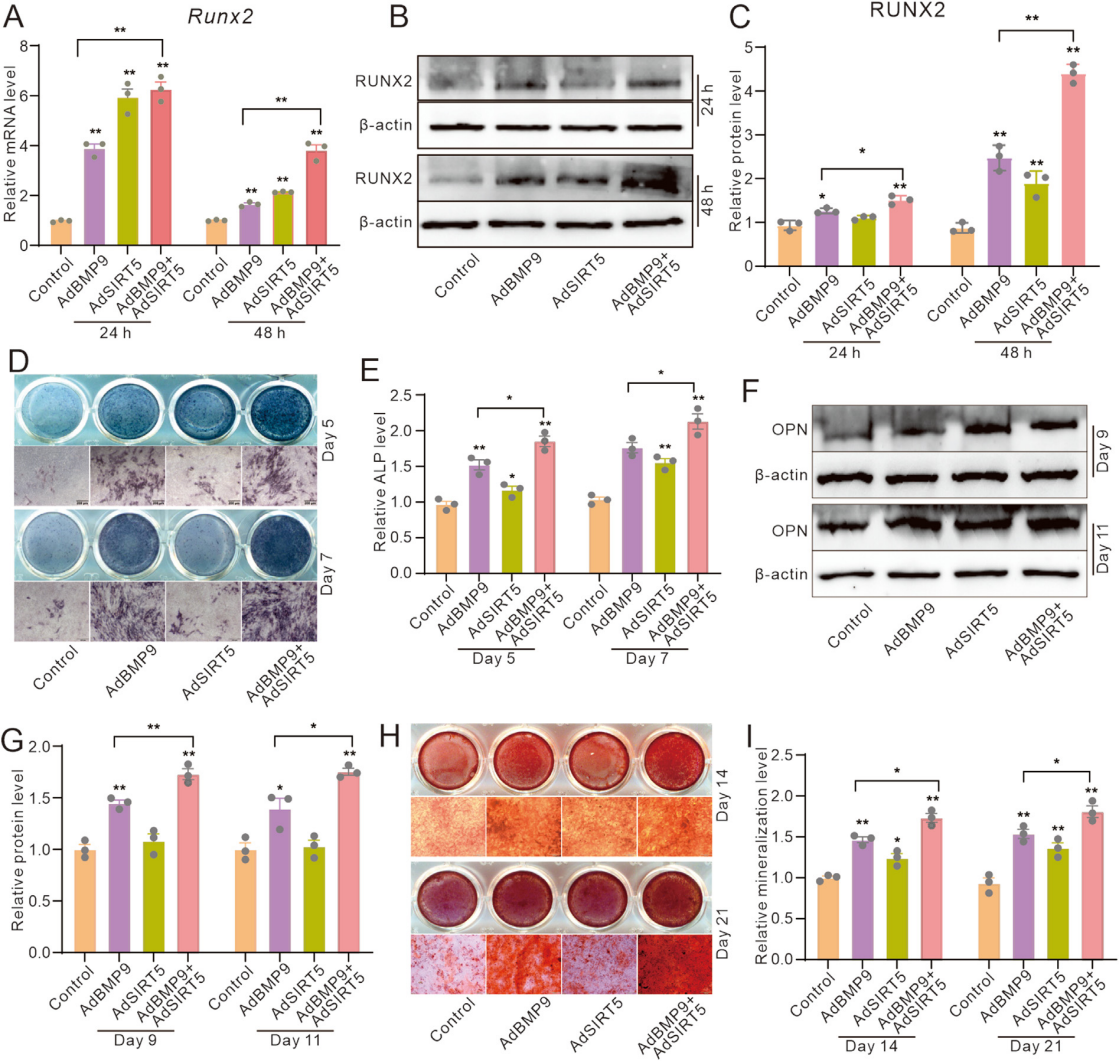
Effects of SIRT5 on the BMP9-induced osteoblastic markers in MEFs. **(A)** Real-time PCR analysis demonstrated the effects of BMP9 and/or SIRT5 on the mRNA expression of RUNX2 in MEFs. **(B)** Western blot assays revealed the influence of BMP9 and/or SIRT5 on the protein levels of RUNX2 in MEFs. **(C)** Quantitative analysis of Western blot data showed the impact of BMP9 and/or SIRT5 on RUNX2 protein abundance in MEFs. **(D)** Histochemical staining illustrated the effects of BMP9 and/or SIRT5 on ALP activity in MEFs. **(E)** Quantitative analysis of histochemical staining data showed the influence of BMP9 and/or SIRT5 on ALP activity in MEFs. **(F)** Western blot assays showed the effects of BMP9 and/or SIRT5 on the protein levels of OPN in MEFs. **(G)** Quantitative analysis of Western blot assay showed the impact of BMP9 and/or SIRT5 on OPN protein abundance in MEFs. **(H)** Histochemical staining showed the effects of BMP9 and/or SIRT5 on mineralization in MEFs. **(I)** Quantitative analysis of histochemical staining showed the influence of BMP9 and/or SIRT5 on mineralization in MEFs. ∗*p* < 0.05 and ∗∗*p* < 0.01 versus control. BMP9, bone morphogenetic protein 9; SIRT5, sirtuin 5; MEFs, mouse embryonic fibroblasts; ALP, alkaline phosphatase.

Subsequently, we determined the effect of SIRT5 on the osteogenic differentiation induced by BMP9 in MEFs. The real-time quantitative PCR and Western blot analyses showed that the expression of RUNX2 was increased by BMP9, which was further enhanced by SIRT5 (Fig. 2A–C). Additionally, ALP activities were increased by BMP9, which was also strengthened by SIRT5 (Fig. 2D, E). Although SIRT5's capacity to elevate OPN protein levels was relatively weaker compared with BMP9, it notably enhanced the effect of BMP9 on OPN (Fig. 2F, G). The mineralization induced by BMP9 was also intensified by SIRT5 (Fig. 2H, I). These results indicated that the osteogenic potential of BMP9 may be enhanced by SIRT5.

### Effects of SIRT5 knockdown on the BMP9-induced osteoblastic markers in MEFs

**Figure 3:**
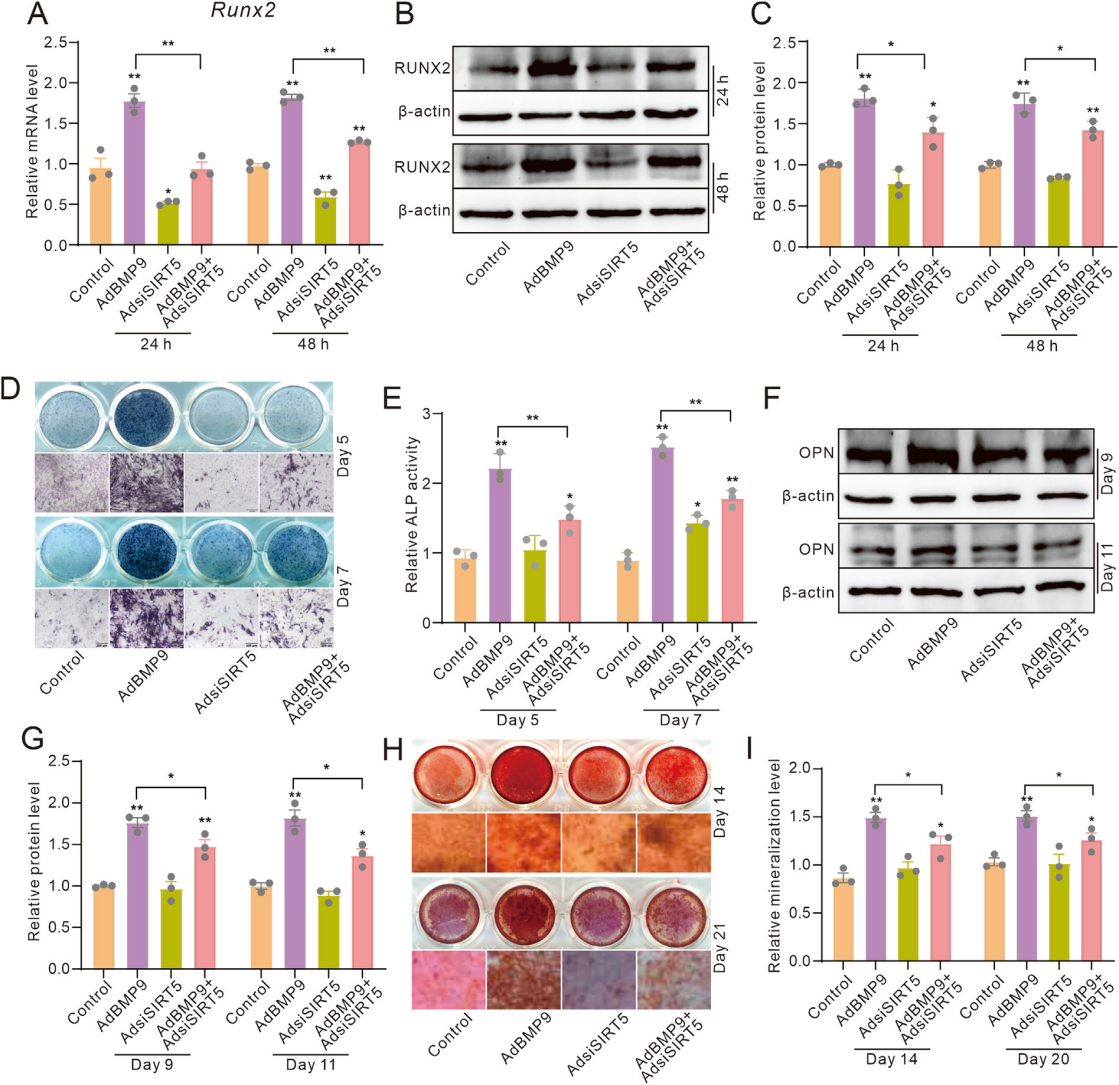
Effects of SIRT5 knockdown on the BMP9-induced osteoblastic markers in MEFs. **(A)** Real-time PCR assay showed the impact of BMP9 and/or SIRT5 knockdown on the mRNA level of RUNX2 in MEFs. **(B)** Western blot assay showed the impact of BMP9 and/or SIRT5 knockdown on the protein level of RUNX2 in MEFs. **(C)** Quantitative analysis of Western blot assay showed the impact of BMP9 and/or SIRT5 knockdown on the protein level of RUNX2 in MEFs. **(D)** Histochemical staining showed the impact of BMP9 and/or SIRT5 knockdown on ALP activities in MEFs. **(E)** Quantitative analysis of histochemical staining showed the impact of BMP9 and/or SIRT5 knockdown on ALP activities in MEFs. **(F)** Western blot assay showed the impact of BMP9 and/or SIRT5 knockdown on the protein level of OPN in MEFs. **(G)** Quantitative analysis of Western blot assay showed the impact of BMP9 and/or SIRT5 knockdown on the protein level of OPN in MEFs. **(H)** Histochemical staining showed the impact of BMP9 and/or SIRT5 knockdown on mineralization in MEFs. **(I)** Quantitative analysis of histochemical staining showed the impact of BMP9 and/or SIRT5 knockdown on mineralization in MEFs. ∗*p* < 0.05 and ∗∗*p* < 0.01 versus control. BMP9, bone morphogenetic protein 9; SIRT5, sirtuin 5; RUNX2, RUNX family transcription factor 2; ALP, alkaline phosphatase; OPN, osteopontin; MEFs, mouse embryonic fibroblasts.

Given that SIRT5 may enhance the BMP9-induced osteogenic differentiation, we subsequently examined whether SIRT5 knockdown could reduce this function of BMP9. Real-time quantitative PCR and Western blot assays showed that the mRNA and protein levels of RUNX2 were reduced by SIRT5 knockdown, thereby dampening the BMP9-induced up-regulation of RUNX2 (Fig. 3A–C). Although SIRT5 knockdown did not significantly reduce ALP activity, it significantly attenuated the BMP9-induced ALP activities (Fig. 3D, E). Additionally, OPN protein level showed no obvious change when treated with SIRT5 knockdown, but the BMP9-stimulated OPN protein level was prominently reduced by SIRT5 knockdown (Fig. 3F, G). The alizarin red S staining results further demonstrated a substantial decrease in BMP9-induced mineralized nodule formation upon SIRT5 knockdown (Fig. 3H, I). These results indicated the pivotal positive role of SIRT5 in regulating the BMP9 osteogenic potential.

### Effects of SIRT5 and/or BMP9 on the expression of HIF-1α in MEFs

**Figure 4:**
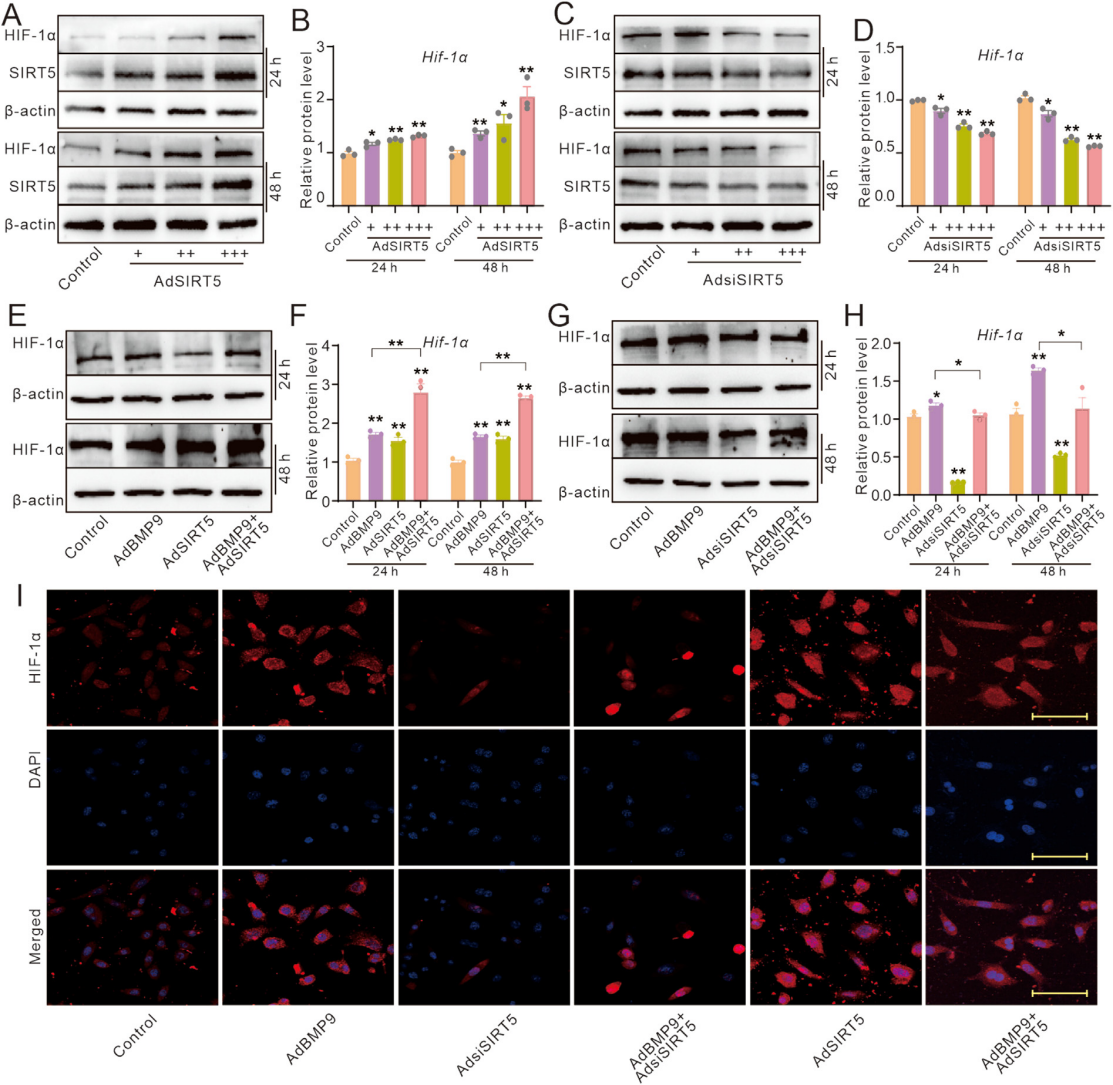
Effects of SIRT5 and/or BMP9 on the protein level of HIF-1α in MEFs. (A) Western blot assay shows the impact of BMP9 and/or SIRT5 on the protein level of HIF-1α in MEFs. (B) Quantitative analysis of western blot assay shows the impact of BMP9 and/or SIRT5 on the protein level of HIF-1α in MEFs. (C) Western blot assay shows the impact of SIRT5 knockdown on the protein level of HIF-1α in MEFs. (D) Quantitative analysis of western blot assay shows the impact of SIRT5 knockdown on the protein level of HIF-1α in MEFs. (E) Western blot assay shows the impact of BMP9 and/or SIRT5 on the protein level of HIF-1α in MEFs. (F) Quantitative analysis of western blot assay shows the impact of BMP9 and/or SIRT5 on the protein level of HIF-1α in MEFs. (G) Western blot assay shows the impact of BMP9 and/or SIRT5 knockdown on the protein level of HIF-1α in MEFs. (H) Quantitative analysis of western blot assay shows the impact of BMP9 and/or SIRT5 knockdown on the protein level of HIF-1α in MEFs. (I) Immunofluorescent staining and confocal assay show the impact of BMP9 and/or SIRT5 knockdown on the protein level of HIF-1α in MEFs. (Scale bar = 40 μm “∗” *P*<0.05, “∗∗” *P*<0.01 vs control. BMP9, bone morphogenetic protein 9; SIRT5, sirtuin 5; MEFs, mouse embryonic fibroblasts; HIF-1α, hypoxia-inducible factor 1 subunit alpha.)

HIF-1α is another crucial factor known to regulate the osteogenic potential of BMP9, yet it is unclear whether HIF-1α can mediate the effect of SIRT5 on this function of BMP9. Thus, we determined the effect of SIRT5 on the expression of HIF-1α. The Western blot and PCR results showed that SIRT5 elevated the protein and mRNA level of HIF-1α (Fig. 4A, B), which was reduced by SIRT5 knockdown (Fig. 4C, D). The Western blot and PCR assay results showed that BMP9 increased the protein and mRNA level of HIF-1α, and SIRT5 had no significant impact on it, but the effect of BMP9 on increasing the expression of HIF-1α was significantly enhanced by SIRT5 (Fig. 4E, F). Conversely, SIRT5 silencing substantially reduced the effect of BMP9 on up-regulating HIF-1α (Fig. 4G, H). Further immunofluorescent staining results showed that SIRT5 enhanced the effect of BMP9 on inducing HIF-1α (Fig. 4I). These results implied that HIF-1α may be involved in mediating the SIRT5 effect on enhancing BMP9 osteogenic potential.

### Effects of SIRT5 and/or HIF-1α on BMP9-induced osteoblastic markers in MEFs

**Figure 5:**
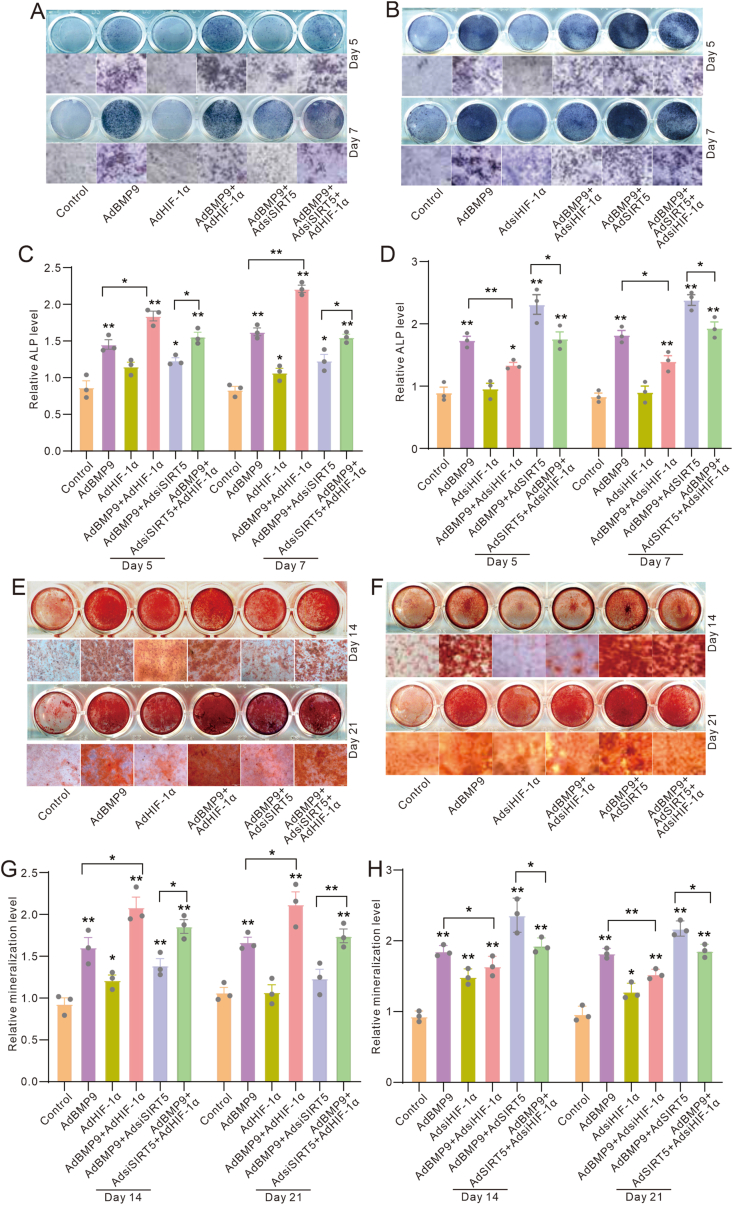
Effects of SIRT5 and/or HIF-1α on BMP9-induced osteoblastic markers in MEFs. (A) Histochemical staining assay shows the impact of BMP9, SIRT5 knockdown, and/or HIF-1α on the ALP activities in MEFs. (B) Histochemical staining assay shows the impact of BMP9, SIRT5, and/or HIF-1α knockdown on the ALP activities in MEFs. (C) Quantitative analysis of histochemical staining assay shows the impact of BMP9, SIRT5 knockdown, and/or HIF-1α on the ALP activities in MEFs. (D) Quantitative analysis of histochemical staining assay shows the impact of BMP9, SIRT5, and/or HIF-1α knockdown on the ALP activities in MEFs. (E) Histochemical staining assay shows the impact of BMP9, SIRT5 knockdown, and/or HIF-1α on the mineralization in MEFs. (F) Histochemical staining assay shows the impact of BMP9, SIRT5, and/or HIF-1α knockdown on the mineralization in MEFs. (G) Quantitative analysis of histochemical staining assay shows the impact of BMP9, SIRT5 knockdown, and/or HIF-1α on the mineralization in MEFs. (H) Quantitative analysis of histochemical staining assay shows the impact of BMP9, SIRT5, and/or HIF-1α knockdown on the mineralization in MEFs. ∗*p* < 0.05 and ∗∗*p* < 0.01 versus control. BMP9, bone morphogenetic protein 9; SIRT5, sirtuin 5; ALP, alkaline phosphatase; HIF-1α, hypoxia-inducible factor 1 subunit alpha; MEFs, mouse embryonic fibroblasts.

Next, we delved into the potential impact of SIRT5 and/or HIF-1α on the BMP9 osteogenic potential. ALP activity assay results showed that HIF-1α potentiated the BMP9-induced ALP activities, whereas SIRT5 knockdown dampened this effect of BMP9; notably, SIRT5 silencing partially suppressed the HIF-1α effect on enhancing the BMP9-induced ALP activities (Fig. 5A–C). Conversely, HIF-1α knockdown significantly reduced the BMP9-induced ALP activities, but SIRT5 increased this effect of BMP9; additionally, SIRT5 rescued the inhibitory effect of HIF-1α knockdown on BMP9-induced ALP activities (Fig. 5B–D). Alizarin red S staining results showed that SIRT5 knockdown reduced the HIF-1α mediated promotion of BMP9-induced mineralization (Fig. 5E–G), while SIRT5 reduced the inhibitory effect of HIF-1α knockdown on BMP9-induced mineralization (Fig. 5F–H). These results suggested that the SIRT5 effect on enhancing BMP9 osteogenic potential may be partially mediated by increasing the level of HIF-1α.

### Effects of SIRT5 and/or HIF-1α on BMP9-induced bone defect repair

**Figure 6:**
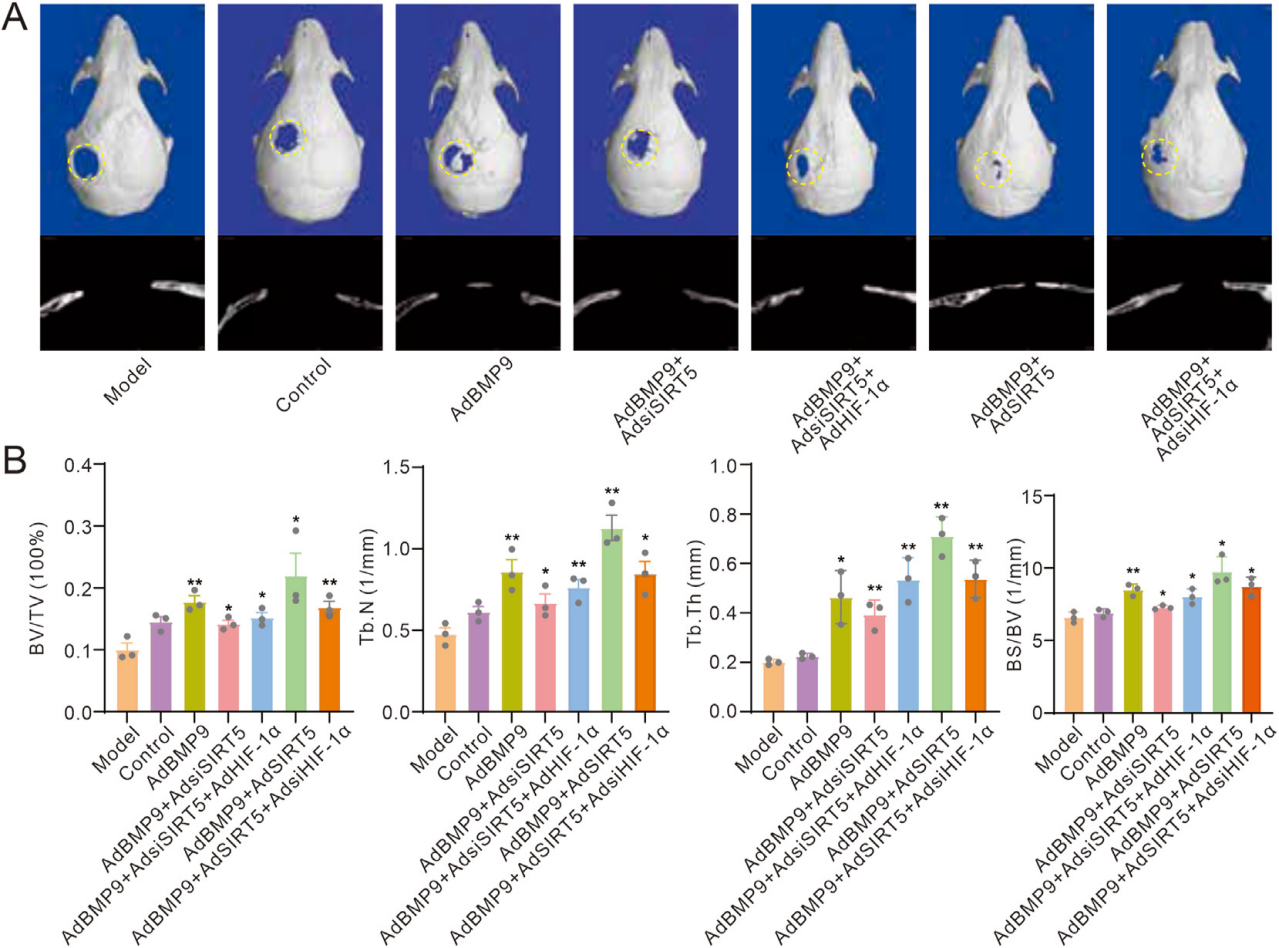
Effects of SIRT5 and/or HIF-1α on BMP9-induced bone defect repair. **(A)** Reconstruction of μ-CT scanning showed the impact of BMP9, SIRT5 knockdown, SIRT5, HIF-1α, and/or HIF-1α knockdown in bone defect repair in mice. **(B)** Quantitative analysis of μ-CT scanning showed the impact of BMP9, SIRT5 knockdown, SIRT5, HIF-1α, and/or HIF-1α knockdown in bone defect repair in mice. ∗*p* < 0.05 and ∗∗*p* < 0.01 versus control. BMP9, bone morphogenetic protein 9; SIRT5, sirtuin 5; HIF-1α, hypoxia-inducible factor 1 subunit alpha.

Next, we proceeded with *in vivo* experimentation to determine the effect of SIRT5 and/or HIF-1α on BMP9-induced bone defect repair. The cranial defect repair model assay results showed that BMP9 accelerated repair processes compared with the control group. Notably, the effect of BMP9 on accelerating repair was inhibited partially when SIRT5 was silenced, which could be obviously rescued by exogenous HIF-1α. Conversely, SIRT5 strengthened BMP9-induced defect repair, which was significantly attenuated by HIF-1α knockdown (Fig. 6A, B). These results confirmed the possible crucial role of HIF-1α in mediating the SIRT5 effect on promoting the osteogenic capability of BMP9.

### Effects of SIRT5 and/or BMP9 on acetylation and malonylation of HIF-1α in MEFs

**Figure 7:**
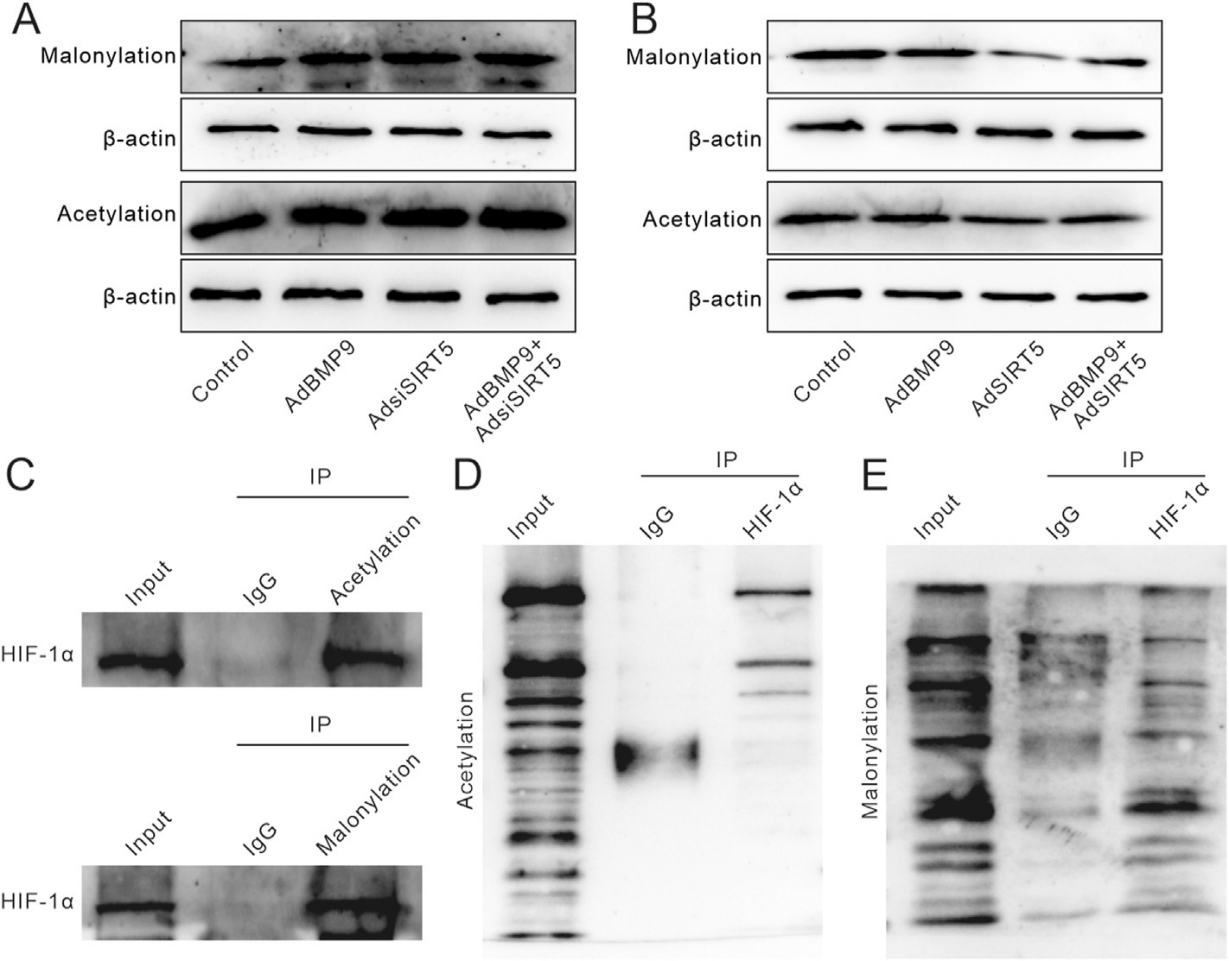
Effects of SIRT5 and/or BMP9 on acetylation and malonylation of HIF-1α in MEFs. **(A)** Western blot assay showed the impact of BMP9 and/or SIRT5 knockdown on the level of global malonylation and acetylation in MEFs. **(B)** Western blot assay showed the impact of BMP9 and/or SIRT5 on the level of global malonylation and acetylation in MEFs. **(C)** Immunoprecipitation and Western blot assay showed the malonylation and acetylation modification of HIF-1α. **(D, E)** Immunoprecipitation and Western blot assay showed the malonylation and acetylation modification of HIF-1α. BMP9, bone morphogenetic protein 9; SIRT5, sirtuin 5; HIF-1α, hypoxia-inducible factor 1 subunit alpha; MEFs, mouse embryonic fibroblasts.

As SIRT5 acts as a de-acylase for post-transcription modification, we next determined its effect on protein modification of HIF-1α, including acetylation and malonylation. Western blot analysis showed that SIRT5 knockdown slightly elevated the global levels of malonylation and acetylation, and enhanced the BMP9's strengthening effects on these modifications (Fig. 7A). Conversely, SIRT5 reduced the BMP9 effect on increasing malonylation and acetylation levels (Fig. 7B). Immunoprecipitation and Western blot assay results showed that HIF-1α underwent both acetylation and malonylation modification (Fig. 7C–E). These data suggested that the effect of SIRT5 on enhancing BMP9 osteogenic potential may partially stem from reducing the levels of HIF-1α acetylation and malonylation.

### Effects of SIRT5 and/or BMP9 on the degradation of HIF-1α in MEFs

**Figure 8:**
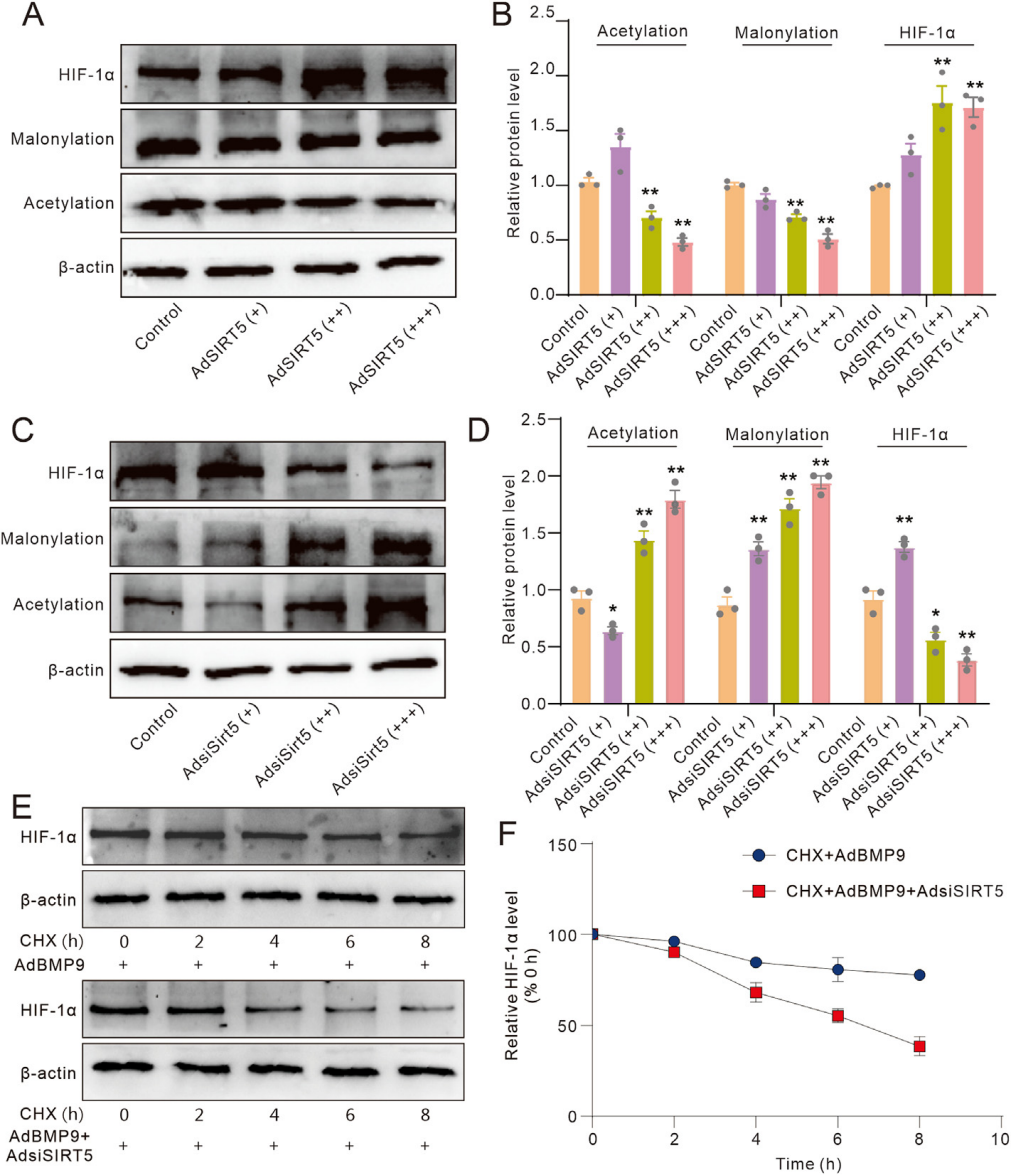
Effects of SIRT5 and/or BMP9 on the degradation of HIF-1α in MEFs. **(A)** Western blot assay showed the effect of SIRT5 on the level of HIF-1α, and global malonylation and acetylation in MEFs. **(B)** Quantitative analysis of Western blot assay showed the effect of SIRT5 on the level of HIF-1α, and global malonylation and acetylation in MEFs. **(C)** Western blot assay showed the effect of SIRT5 knockdown on the level of HIF-1α, and global malonylation and acetylation in MEFs. **(D)** Quantitative analysis of Western blot assay showed the effect of SIRT5 knockdown on the level of HIF-1α, and global malonylation and acetylation in MEFs. **(E)** Western blot assay showed the effect of SIRT5 knockdown and/or BMP9 on the degradation of HIF-1α. **(F)** Quantitative analysis of Western blot assay showed the effect of SIRT5 knockdown and/or BMP9 on the degradation of HIF-1α. ∗*p* < 0.05 and ∗∗*p* < 0.01 versus control. BMP9, bone morphogenetic protein 9; SIRT5, sirtuin 5; CHX, cycloheximide; HIF-1α, hypoxia-inducible factor 1 subunit alpha; MEFs, mouse embryonic fibroblasts.

Finally, we determined whether SIRT5 could affect the protein stability of HIF-1α. Western blot analyses showed that SIRT5 reduced global acetylation and malonylation levels while simultaneously elevating the level of HIF-1α (Fig. 8A, B). Conversely, SIRT5 knockdown led to an increase in global acetylation and malonylation levels, accompanied by a substantially decreased HIF-1α level (Fig. 8C, D). Further Western blot assay results showed that SIRT5 knockdown accelerated the degradation of HIF-1α (Fig. 8E, F). These results indicated that SIRT5 may keep the stability of HIF-1α by reducing its acetylation and malonylation level at least.

## Discussion

BMP9 and a few other members of the BMP family possess excellent osteo-induction functions, which need to be further enhanced due to some defects. In this study, we determined the role of SIRT5 in BMP9-induced osteogenic differentiation in mesenchymal stem cells and the possible mechanism for this function. We found that SIRT5 was up-regulated by BMP9, and the BMP9-induced osteogenic differentiation and bone defect repair were enhanced by SIRT5 but reduced by silencing SIRT5. The SIRT5 effect on increasing BMP9 osteogenic potential was partially mediated by reducing acetylation and malonylation levels of HIF-1α. This finding enlarges our vision of how BMP9 induces osteogenic differentiation in mesenchymal stem cells and may provide another potent target to strengthen BMP9 osteoblastic potential.

BMP was first found by Dr. Urist for its ability to promote bone formation.^19^ BMP9 was first identified in the fetal mouse liver cDNA library, which has been demonstrated as the most competent osteogenic BMP member.^20^^,^^21^ In addition to osteogenic induction, BMP9 is also involved in various biological functions, such as inflammation, glucose and lipid homeostasis, and angiogenesis.^22^, ^23^, ^24^ In bone development and repair, BMP9 may be a promising factor.^21^ Although it shows a stronger effect than that of BMP2 and BMP7 to induce osteogenesis, the duration for BMP9-induced bone formation still needs a long time, and adipogenesis can also be induced simultaneously.^3^ Therefore, the osteoblastic-induction ability of BMP9 may still need to be elevated to meet the ideal requirement of bone repair or bone tissue engineering. As reported, BMP9 exerts its function on inducing osteogenic differentiation through canonical BMP/Smad or non-canonical BMP/Smad pathways.^3^^,^^6^ However, the specific mechanisms that contribute to this function still need to be well elucidated, which may contribute to making up its defects in osteogenesis.

The model of glucose metabolism is changed during osteogenic differentiation in mesenchymal stem cells. Usually, glycolysis flux increases during osteogenic differentiation even if there is no change in oxidative phosphorylation flux.^25^ The osteogenic potential will be reduced if glycolysis flux is reduced.^26^ Thus, increasing glycolysis may be a possible strategy to strengthen the osteogenic potential of BMP9. Our previous study demonstrated that PDK4 could promote this effect of BMP9, which may be due to an increase in the glycolysis flux partially.^27^ As reported, the product of glycolysis, lactic acid, also shows a promoting effect on osteogenesis.^28^^,^^29^ It is well known that BMP9 plays an important role in regulating the metabolism of glucose, which may contribute to regulating the osteo-induction potential of BMP9, but the concrete mechanism remains unclear. The metabolism of glucose is well coordinated by various factors. SIRTs are NAD^+^-dependent conserve proteins, include seven members, and are located in the cytoplasm, mitochondria, or nucleus. These proteins are involved in the regulation of various physiological processes by acting as acetylase or monoADP ribosyltransferase.^30^ Several SIRTs are involved in glucose metabolism, such as SIRT3, SIRT6, and SIRT5.^17^^,^^31^^,^^32^ SIRT5 is another lysine deacetylase that is located primarily in mitochondria and mainly catalyzes the deglutarylation, desuccinylation, demalonylation, and deacetylation of lysine residual. In this way, SIRT5 takes part in various physiological processes, such as metabolism and detoxification of reactive oxygen species.^33^ It was reported that SIRT6 could promote osteogenic differentiation through BMP signaling, and SIRT1 could enhance the BMP9-induced osteogenesis and angiogenesis in mesenchymal stem cells or promote osteoporotic bone healing.^34^, ^35^, ^36^ SIRT3 deficiency results in osteoblast dysfunction and obvious osteopenia, but exogenous SIRT3 can reverse these changes.^37^ In contrast, Li et al found that SIRT6 could protect vascular smooth muscle cells from osteogenic trans-differentiation in chronic kidney disease.^38^ Thus, SIRTs may contribute to regulating the balance of the skeletal system. SIRT5 can attenuate compression-induced intervertebral disc degeneration via desuccinylation of AIFM1.^39^ Besides, SIRT5 can also contribute to bone remodeling by desuccinylation modification of Cdc42.^16^ To date, it remains unknown whether SIRT5 is involved in the BMP9-induced osteogenic differentiation and up-regulation of HIF-1α. In this study, we found that SIRT5 was up-regulated by BMP9 substantially in MEFs. The BMP9-induced osteogenic markers were increased by exogenous SIRT5 but reduced by silencing SIRT5. Here, SIRT5 may contribute to the BMP9-induced osteoblastic differentiation.

As reported, aerobic glycolysis influx increased during osteogenic differentiation of mesenchymal stem cells. This suggests that osteogenesis can be affected by the level of energy supply. SIRT5 can remove succinyl of lysin residual and plays an important role in keeping energy homeostasis.^40^ Oxygen consumption rate is associated with energy supply, and hypoxia can decrease the energy supply. The most important transcriptional factor HIF-1α can be increased to protect cells from being damaged from hypoxic conditions. In addition, lactate can promote osteoblast differentiation by stabilizing HIF-1α.^29^ Thus, there may exist an interaction between SIRT5 and HIF-1α to keep energy homeostasis synergistically. Previous studies have demonstrated that HIF-1α can promote BMP9-induced osteogenic differentiation and vascularization, and BMP9 can also up-regulate HIF-1α.^41^^,^^42^ For this evidence, we speculated that the effect of SIRT5 on promoting the osteogenic potential of BMP9 may be mediated by increasing the level of HIF-1α. In this study, we found that the effect of BMP9 on increasing HIF-1α protein level was enhanced by exogenous SIRT5, but reduced by silencing SIRT5. The BMP9-induced osteogenic markers were reduced by silencing SIRT5 which were mostly restored by exogenous HIF-1α. In contrast, the effect of SIRT5 on increasing the level of osteogenic markers induced by BMP9 was attenuated obviously by silencing HIF-1α. The bone defect repair assay recaptured the same results. Hence, HIF-1α may mediate the SIRT5 effect on promoting the osteo-induction potential of BMP9. As reported, the half-life of HIF-1α is very short, which determines the physiological function of HIF. Thus, the stability of HIF-1α is very important for hypoxia stress.^29^ SIRT5 acts as a deacetylase to remove acetyl, malonyl, or succinyl from the lysin residual of the substrate to change their functions. With immunoprecipitation and Western blot assay, we demonstrate that HIF-1α can be modified by acetylation and malonylation, which can be reduced by SIRT5. Besides, SIRT5 can also attenuate the degradation of HIF-1α markedly.

Taken together, SIRT5 can be up-regulated by BMP9, and the BMP9-induced osteoblastic differentiation can also be strengthened by SIRT5. HIF-1α may be modified by the SIRT5-mediated deacetylation and demalonylation, which can keep its stabilization. Our findings may provide another important potent target to strengthen the osteogenic potential of BMP9, which may facilitate the improvement of bone-tissue engineering to meet clinical needs.

## CRediT authorship contribution statement

**Lu Liu:** Methodology, Investigation, Data curation. **Fanglin Ye:** Validation, Methodology, Investigation, Data curation. **Yue Jiang:** Methodology, Formal analysis. **Wenting Liu:** Resources. **Dongmei He:** Validation, Resources. **Wenge He:** Visualization, Methodology. **Xiang Gao:** Resources. **Hang Liu:** Software. **Junyi Liao:** Supervision, Funding acquisition, Conceptualization. **Baicheng He:** Writing – review & editing, Writing – original draft, Project administration, Funding acquisition, Formal analysis. **Fang He:** Writing – review & editing, Writing – original draft, Supervision.

## Ethics declaration

The animal experimentation was approved by the Medical Research Ethics Committee of Chongqing Medical University.

## Data availability

The datasets employed within this work are made available upon request and directed to the corresponding author.

## Funding

The research was financially supported by the 10.13039/501100004374Chongqing Medical University Program for Youth Innovation in Future Medicine (No. W0154 to B.C.H. and J.Y.L.) and 10.13039/501100002865Chongqing Science and Technology Bureau (China) (No. CSTB2024NSCQ-MSX0411 to B.C.H.).

## Conflict of interests

The authors hereby declare the absence of any conflict of interests in relation to this work.
